# Abnormal hippocampal–thalamic white matter tract development and positive symptom course in individuals at ultra-high risk for psychosis

**DOI:** 10.1038/npjschz.2015.9

**Published:** 2015-05-06

**Authors:** Jessica A Bernard, Joseph M Orr, Vijay A Mittal

**Affiliations:** 1 Department of Psychology and Neuroscience, University of Colorado Boulder, Boulder, CO, USA; 2 Institute for Cognitive Science, University of Colorado Boulder, Boulder, CO, USA; 3 Center for Neuroscience, University of Colorado Boulder, Boulder, CO, USA

## Abstract

**Background/Objectives:**

Abnormal development of the hippocampus has been reported in adolescents at ultra-high risk (UHR) for psychosis and thalamic abnormalities have been found. However, the white matter connections between the hippocampus and the thalamus have not been studied. The connections between these regions are of key importance to our understanding of the pathophysiology of psychosis.

**Methods:**

Twenty-six UHR and 21 healthy age-matched controls were tested at a baseline assessment and 12 months later. Symptoms were assessed at both the time points and all the participants underwent diffusion tensor imaging scans. We used tractography to trace the white matter connections in each individual between the thalamus and hippocampus and then extracted fractional anisotropy (FA) to assess white matter structural integrity.

**Results:**

There was a significant group by time interaction indicating that FA decreased in UHR, and increased in controls over 12 months. Across both groups, baseline FA of the thalamic–hippocampal tract was predictive of positive symptoms at 12-month follow-up. Critically, this pattern remained significant in UHR individual group alone. At baseline, those with higher FA, indicative of abnormal white matter development, show higher positive symptoms 1 year later.

**Conclusions:**

Here, we provide evidence to indicate that there are differences in white matter development in hippocampal–thalamic connections, both of which are important nodes in networks associated with schizophrenia. Furthermore, abnormal developmental patterns in UHR individuals are associated with positive symptom course.

## Introduction

The hippocampus and thalamus are subcortical regions involved in a wide array of cognitive functions and both structures share direct white matter connections.^1^ Furthermore, patients with schizophrenia show decreased volume in the thalamus,^2^ and both hippocampal volume and dysfunction are related to memory deficits and positive sypmtoms.^3–5^ Importantly, these regions and their associated circuitries have been postulated to be especially important for the development of psychosis.^6,7^ However, to date, there have been no longitudinal investigations examining the tracts connecting these two structures in the ultra-high risk (UHR) period.

Schizophrenia has been conceptualized as a disorder of network dysfunction.^8^ Investigating connections between key nodes associated with the disease is especially important. One way to do so is using diffusion tensor imaging (DTI) tractography. This method allows for investigators to carefully trace the white matter connections between specific regions within the brain.^1^ White matter integrity (fractional anisotropy; FA) within specific tracts can then be examined. Patients with schizophrenia have altered white matter integrity associated with both the hippocampus and thalamus and their cortical connections.^9–12^ Furthermore, abnormal white matter development in UHR populations has been reported as compared with healthy controls.^13,14^ However, open questions remain. Though direct connections between both the thalamus and hippocampus exist anatomically,^1^ they have not been mapped using tractography in psychosis. To better understand the development of psychosis, longitudinally investigating the hippocampal–thalamic tract in UHR individuals with respect to symptom severity is crucial.

In UHR, populations between 15–35% of individuals will go on to develop a psychotic disorder.^15–17^ Investigating this population is important for our understanding of the development of psychosis, and these individuals are also often free from confounds (e.g., medications, drug/alcohol abuse) that are common in patients with schizophrenia. Within UHR samples, there is evidence indicating volumetric differences in the hippocampus and thalamus relative to controls.^18–23^ It has also been suggested that hippocampal volume decreases with disease progression from the at-risk state to the first episode of psychosis.^24^ Furthermore, both patients with schizophrenia and their siblings have decreased white matter structural integrity in the hippocampus^9^ and in the cortex,^13,25^ though mixed findings have been reported.^26^ In a population of UHR individuals, in those that converted to psychosis there were areas of both increased and decreased white matter volume^27^ suggesting that white matter development in UHR individuals may not be as simple as linear changes in one direction. Finally, recent work investigating white matter development has indicated abnormal developmental patterns in UHR individuals.^13,14^ However, this work did not utilize tractography approaches. Given the hippocampal–thalamic connections, the evidence to indicate altered structure and function in both schizophrenia and UHR populations, and their links to positive symptomatology, investigating hippocampal–thalamic white matter connections in UHR populations may be especially informative for our understanding of the development of psychosis.

Here, a group of UHR individuals and healthy controls underwent DTI at two time points. We are the first to take a more intensive tractography approach to investigate white matter development in the hippocampal–thalamic tract in UHR individuals. Consistent with our prior work investigating white matter development in UHR individuals,^14^ we hypothesized that there would be abnormal white matter development in the UHR group. Further, we hypothesized that baseline FA would be associated with positive symptoms 12 months later, given prior work suggesting a role for these regions in the development of psychosis,^6,7^ and their associations with positive symptom severity.^3–5^ There is mixed evidence regarding hippocampal volume in UHR populations, with some indication that volume may be larger.^28^ This may be, in part, due to more white matter in the region, manifest in greater FA. Finally, prior work has found higher FA in siblings of schizophrenia patients.^26^ Thus, we predicted that higher FA, indicative of pathophysiology at baseline, would be associated with more severe positive symptoms 12 months later.

## Materials and Methods

### Participants

Twenty-six UHR (9 female) and 21 control (13 female) individuals between 12 and 21 years of age were recruited as part of a larger study to the Adolescent Development and Preventative Treatment research program at the University of Colorado Boulder. This longitudinal investigation aims to better understand the development of psychosis. Table 1 provides demographic information for all the participants. Exclusion criteria for both groups included history of head injury, the presence of a neurological disorder, life-time substance dependence (as assessed by the Structured Clinical Interview for Axis I DSM-IV Disorders)^29^ and the presence of any contraindication to the magnetic resonance imaging environment. For UHR participants, we also excluded those with an Axis I psychotic disorder. In control subjects, the presence of an Axis I disorder or a psychotic disorder in first-degree relatives was an exclusion criterion. All the procedures were approved by the University of Colorado Institutional Review Board. Before beginning the study, all the participants signed an IRB-approved consent form. In individuals younger than age 18, a parent or guardian provided consent, and the participant provided assent.

### Symptom assessment

The SIPS (Structured Interview for Prodromal Syndromes)^30^ was administered to all participants to diagnose a prodromal syndrome. The SIPS measures distinct categories of prodromal symptom domains including positive and negative dimensions and is scored from 0 to 6 for each symptom. We looked at the total positive and negative symptom scores. All the UHR participants in this study met the criteria for a prodromal syndrome. This was defined by moderate levels of positive symptoms (a SIPS score of 3–5 in one or more of the five positive symptom categories; Table 1), and/or a decline in global functioning in association with the presence of schizotypal personality disorder, and/or a family history of schizophrenia.^30^ All interviewers had inter-rater reliabilities that exceeded Kappa ⩾80.

### Neuroimaging

Magnetic resonance imaging scans were acquired using a 3-Tesla Siemens Tim Trio magnetic resonance imaging scanner (Siemens AG, Munich, Germany) using a standard 12-channel head coil. Structural images were acquired with a T1-weighted 3D magnetization prepared rapid gradient multi-echo sequence (sagittal plane; repetition time (TR)=2,530 ms; echo times (TE)=1.64 ms, 3.5 ms, 5.36 ms, 7.22 ms, 9.08 ms; GRAPPA parallel imaging factor of 2; 1 mm^3^ isomorphic voxels, 192 interleaved slices; FOV=256 mm; flip angle=7°; time=6:03 min). Structural connectivity was assessed with a diffusion-weighted scan (71 gradient directions; TR=9,600 ms; TE=86 mm; GRAPPA parallel imaging factor 2; β-value=1,000 s/mm^2^; FOV=256 mm; 72 slices; 2 mm^3^ isomorphic voxels; seven β0 images).

### DTI data processing

**Figure 1:**
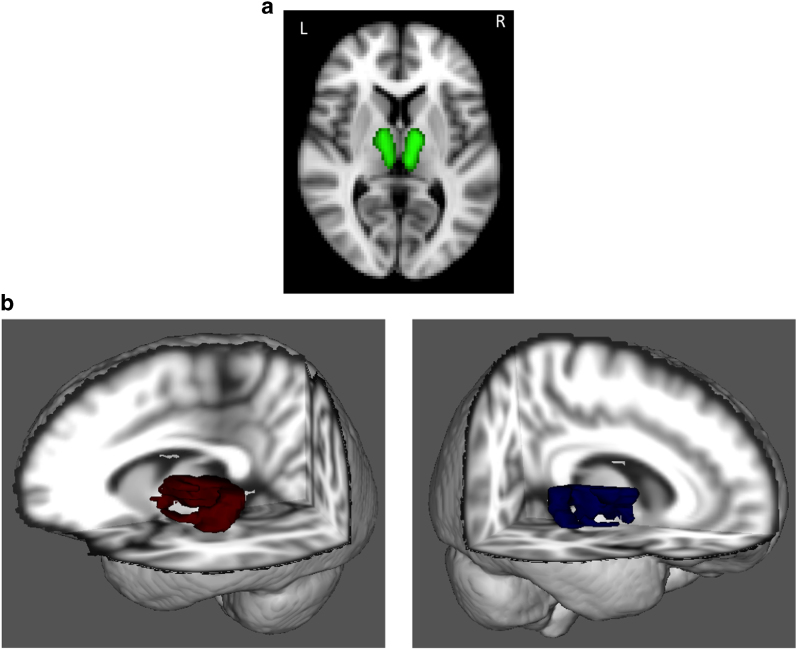
(**a**) Seed regions where we started tractography (coronal slice, *z*=39) in the left and right hemisphere shown in green. Both hemispheres were seeded separately in our analyses. (**b**) The left (red) and right (blue) hippocampal–thalamic white matter tract masks, mapped using tractography starting in the thalamus. FA in these tracts was extracted from both the UHR and control groups. The tracts extend around the posterior edge of the thalamus and then proceed anteriorly into the hippocampus and temporal lobe consistent with prior work.^1^ FA, fractional anisotropy; UHR, ultra-high risk.

First, we created masks of the left and the right prefrontal region of the thalamus (Figure 1a), defined using the tractography-based segmentation of Johansen-Berg *et al*.^31^ in FSL (FMRIB Software Library). The thalamus masks were thresholded at 10% and then binarized for use in our analyses. Though we were interested in hippocampal–thalamic connections, we used the prefrontal subregion of the thalamus because of the connection overlap between adjacent sub-areas of the thalamus.^31^ There is a large degree of overlap between regions of the thalamus that connect to the prefrontal cortex and the temporal lobe,^1^ which are encompassed in this mask area.

Diffusion weighted images were processed using FSL’s FDT toolbox. Images were first corrected for motion and eddy current distortions. Diffusion parameters were calculated at each voxel, accounting for crossing fibres in two directions using *BEDPOSTX* (Bayesian Estimation of Diffusion Parameters Obtained using Sampling Techniques).^32^ Probabilistic tractography was performed between the left and right thalamus masks and the rest of the brain. All tractography analyses were implemented in FSL using probtrackX. We used a step length of 0.5 mm, with 5,000 streamlines, a fibre threshold of 0.1, and used modified Euler streamlining. Tracking was stopped when the streamline reached the edge of the brain mask, when tracking reached 2,000 steps (equivalent to a distance of 1 m), or when the pathway exceeding ±80 degrees from one step to the next.

All tractography was performed in standard space (Montreal Neurological Institute), using nonlinear transformations between individual subject diffusion space and standard space. To create a group average tract map, each individual subject tract map was divided by the total number of streamlines from the seed mask, thresholded at 10%, and then binarized to create a mask. We summed together the individual masks, and divided by the total number of subjects (i.e., 47) to create the group probability map. This procedure was done for each seed mask. These maps were thresholded so that the tract passed through a given voxel in at least 50% of subjects. These maps were then visualized using FSLView and corrected to include only the tract connecting the thalamus to the hippocampus (Figure 1b). The tracts included in our analyses correspond to those found to connect the thalamus to the temporal lobe through the hippocampal formation, as mapped by Behrens *et al*.^1^ FA was extracted from each tract for all subjects.

### Statistical analyses

All statistical analyses were carried out using SPSS Statistics version 22 (IBM Corporation, Armonk, NY, USA). Demographic variables were compared using independent samples *t*-tests or chi-squared tests. Group differences in white matter development for the left and right hippocampal–thalamic tract were evaluated using 2×2 (group by time) repeated measures analyses of covariance. To control for the effects of development, the analyses controlled for baseline age. We also dummy-coded antipsychotic medication use and controlled for this factor. Each hemisphere was analyzed independently, and statistical significance was set at *P*<0.05. Follow-up analyses of covariance were conducted to further explore these results.

We investigated relationships between baseline FA in the hippocampal–thalamic tract and symptoms 12 months later using hierarchical regression analyses. Positive and negative symptoms at the 12-month follow-up assessment were used as the dependent variables. Negative symptoms were included to test the specificity of the FA-symptom prediction relationships. In the first block, we entered baseline positive or negative symptom severity. In the second block, baseline age and antipsychotic medication status were entered, and in the third block we entered FA of the hippocampal–thalamic tracts. The left and right hemispheres were analyzed independently. We tested the significance of the magnitude of *R*^2^ change (Δ*R*^2^) in symptom prediction. Significance was evaluated using a one-tailed *P*<0.05, given our directional hypotheses regarding baseline FA and symptom severity 12 months later. This analysis allows us to test the hypothesis that baseline FA in the hippocampal–thalamic tract is associated with more severe positive symptoms 12 months later.

## Results

Demographic variables did not differ between the two groups (see Table 1 for statistical results). Though there are more females in the UHR group, a chi-square test did not indicate a significant difference. There were no differences in head motion during the DTI scan at either baseline or at 12-month follow-up (in all cases *P*>0.25). Furthermore, there was no significant difference in antipsychotic usage, though only UHR participants (*n*=3) were taking these medications (risperidrone, abilify, and both abilify and Seroquel). As expected, the UHR group showed significantly higher positive and negative symptoms at both baseline and at 12-month follow-up. At follow-up, two of the participants taking antipsychotics at baseline were no longer taking medications and the remaining individual transitioned from taking risperidrone to taking Seroquel. Two additional participants began taking antipsychotic medications (Seroquel and abilify). Three UHR participants (11.5%) converted to psychosis during the 12 months between the baseline and follow-up assessments, consistent with recent longitudinal studies in UHR populations.^33^

**Figure 2:**
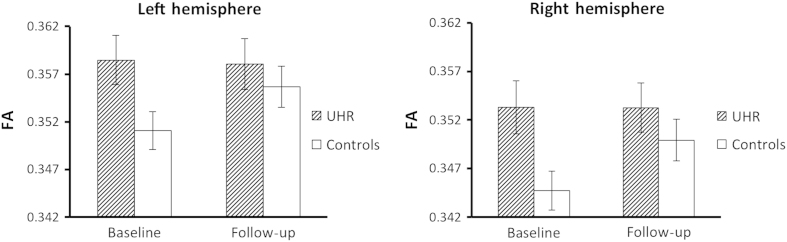
Group by time interactions with respect to FA in the left and right hemispheres. In both cases, the interactions were significant. Follow-up analyses of covariance indicated that UHR individuals show decreased FA in the left hemisphere and are stable in the right, while there are numerical increases in FA in the control group. Error bars represent the standard error of the mean. FA, fractional anisotropy; UHR, ultra-high risk.

With respect to hippocampal–thalamic white matter development, our analyses revealed significant group by time interactions in both the left (F_(1,43)_=9.60, *P*<0.005, *η*^2^_p_=0.183) and right (F_(1,43)_=10.80, *P*<0.005, *η*^2^_p_=0.201) hemispheres (Figure 2). However, there was no significant main effect of group (left: F_(1,43)_=1.59, *P*>0.2, *η*^2^_p_=0.036; right: F_(1,43)_=2.44, *P*>0.1, *η*^2^_p_=0.054), and there was no main effect of time (left: F_(1,43)_=2.61, *P*>0.1, *η*^2^_p_=0.057; right: F_(1,43)_=1.24, *P*>0.2, *η*^2^_p_=0.028). Follow-up analyses of covariance indicated that the significant interaction was likely due to two factors. First, numerically, there were increases in FA in both the left and right hemisphere in the control participants as seen in Figure 2, though this was not statistically significant (left: F_(19)_=0.37, *P*>0.5, *η*^2^_p_=0.019; right: F_(19)_=0.35, *P*>0.5, *η*^2^_p_=0.018). Second, FA in the UHR group remained relatively unchanged over the course of 12 months in the right hemisphere (F_(23)_=0.74, *P*>0.3, *η*^2^_p_=0.031), but in the left hemisphere there was a trend indicating decreased FA over the 12-month period between baseline and follow-up (F_(23)_=2.99, *P*=0.097, *η*^2^_p_=0.=0.115). Though not statistically significant, together, these results are indicative of abnormal patterns of white matter development in the hippocampal–thalamic tracts and are driving the significant interactions.

**Figure 3:**
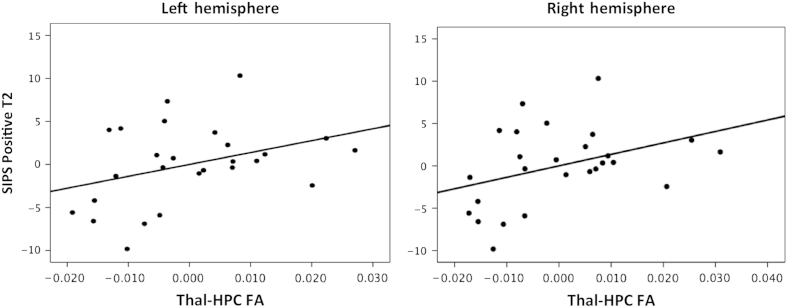
Scatterplots representing the partial correlations of the left hippocampal–thalamic FA (left) and right hippocampal–thalamic FA (right) controlling for baseline symptom levels, age, and antipsychotic medication usage at baseline. In both instances, the addition of the FA variable to the stepwise regression models accounted for a significant increase in the variance explained by the models. FA, fractional anisotropy; SIPS, Structured Interview for Prodromal Syndromes; Thal–HPC, thalamic–hippocampal; UHR, ultra-high risk.

Finally, we were particularly interested in the associations between baseline FA and positive symptoms at 12 months. Baseline FA in the left hemisphere accounts for an additional 7% of the variance in positive symptoms (*β*=0.285, *P*<0.05; Table 2) at 12-month follow-up when accounting for baseline symptoms, antipsychotic medications, and baseline age, whereas in the right hemisphere, an additional 8% of variance was accounted for (*β*=0.299, *P*<0.05). Higher FA at baseline was associated with worse positive symptoms 12 months later. Scatterplots showing the association between baseline FA and follow-up positive symptoms are presented in Figure 3. Finally, to investigate the specificity of these relationships, we investigated negative symptom severity. FA at baseline did not significantly account for any additional variance in negative symptom severity at 12-month follow-up (Table 2). The results remain the same when we remove the three individuals taking antipsychotic medications from our analyses.

## Discussion

Using sophisticated tractography methods targeting hippocampal–thalamic white matter connections, we found evidence indicative of abnormal white matter development in UHR individuals. Broadly speaking, this is in support of neurodevelopmental and network theories of schizophrenia. Over 12 months, our data indicate abnormal white matter development in UHR individuals, as seen in prior longitudinal work in prodromal samples.^13,34^ Consistent with prior single-time-point DTI studies implicating white matter abnormalities in UHR individuals,^13,35,36^ our longitudinal approach extends these findings to include hippocampal–thalamic connections, and both structures have been implicated in schizophrenia and UHR populations.^14,18–22,24^ Furthermore, we demonstrated that baseline FA in the hippocampal–thalamic tract is specifically correlated with positive symptom severity 12 months later. This latter finding indicates the potential for neural measures to predict disease course in UHR populations.

Somewhat surprisingly, our findings indicate that UHR individuals have higher FA at baseline in the hippocampal–thalamic tract. The majority of prior work investigating patients with schizophrenia and their siblings have indicated decreased FA in the hippocampus,^9^ and more broadly, disrupted white matter connections have been described in schizophrenia patients,^11,12,37–39^ though mixed results indicating higher FA or more white matter have been reported.^26,27^ Crucially, our findings are indicative of abnormal development. Our follow-up analyses demonstrated that there was a trend indicating decreases in FA in the UHR group over 12 months. Higher FA in this tract seems to be abnormal, and indeed it is higher baseline FA that positively predicts more severe positive symptoms 12 months later. This is also consistent with some volumetric evidence that has suggested that the hippocampus may be larger in UHR individuals.^20,40^ White matter may be driving these findings of larger hippocampal volume. However, this does differ from the broader literature, which indicates decreased white matter structural integrity in UHR populations.

The associations between baseline FA in the hippocampal–thalamic tract and positive symptoms 12 months later are consistent with literature indicating that these regions are associated with positive symptom severity.^3–5^ Our findings, specific to positive symptoms, extend this literature to include UHR individuals and provide a developmental perspective. Given that these two subcortical regions are postulated to be important in the development of psychosis,^6,7^ it is perhaps not surprising that integrity in their white matter connections is predictive of positive symptom course. This is consistent with the network perspective of schizophrenia, and suggests that a common developmental factor that acts on this network may be underlying this finding. Future work investigating this tract and its development, along with targeted interventions that focus on the hippocampus and thalamus will be especially informative for our understanding of the development of psychosis.

Although our results provide key new findings regarding hippocampal–thalamic white matter connections, particularly with respect to positive symptom course, there are several limitations to consider. First, individuals were only investigated over 12 months, and while increasing positive symptoms can indicate a poor course of illness, we did not assess conversion to psychosis (only three participants had received a conversion diagnosis at 12 months). Continued follow-ups are necessary, and this work is ongoing. In addition, larger samples will likely result in more individuals who convert to psychosis, and the ability of baseline FA to predict conversion to psychosis can be assessed. Within our sample, several participants were taking antipsychotic medications at baseline and 12-month follow-up. Though only a small percentage of the sample was taking neuroleptics during this investigation, understanding the influence of these medications on neurodevelopment is critical, and future studies with larger samples will be integral for examining this question. Relatedly, larger samples that are more equally matched on sex are also needed. There are more males than females in our UHR group, doubling the male control sample. Given the developmental nature of this study, it may be that there are effects of sex impacting these findings. Indeed a recent review from Peters and Karlsgodt^41^ noted that testosterone is an important factor in white matter development in adolescent males, and may be driving the higher FA values seen at baseline in our sample given that there are more males in the UHR group. Future work with matched samples is necessary to tease apart the effects of disease and sex on white matter development in this important population. Finally, recent work in nonhuman primates has called into question the accuracy of tractography methods for delineating white matter tracts in the brain.^42^ With that said, more detailed methods that are used in animal models are not feasible for use in humans. Thus, tractography remains a useful and important tool for measuring white matter connections in the human brain.^42^

Together, we have provided additional evidence in support of neurodevelopmental models of schizophrenia, and have demonstrated a relationship between hippocampal–thalamic white matter and positive symptom course. These important new findings advance our understanding of the potential role of this subcortical circuit in the pathophysiology of schizophrenia.

## Figures and Tables

**Table 1:** Sample information and demographic characteristics of the UHR and control groups (mean and standard deviation)

	*UHR*	*Controls*	*95% CI*
Males	17	8	—
Females	9	13	—
Age (years)	18.73 (1.78)	17.71 (2.65)	−0.288–2.32
Parental education (years)	16.46 (2.13)	16.19 (2.41)	−1.08–1.62
Baseline antipsychotic medication (*N*)	3	0	—
			
*Baseline symptoms*			
Positive***	12.23 (3.65)	0.67 (1.28)	9.88–13.25
Negative***	12.12 (7.04)	0.48 (0.81)	8.52–14.76
			
*Follow-up symptoms*			
Positive***	11.73 (6.41)	0.05 (0.22)	8.86–14.51
Negative***	8.69 (6.97)	0.24 (0.54)	5.38–11.53

Abbreviations: CI, confidence interval; UHR, ultra-high risk.

***P<0.001, two-tailed. 95% CI (upper bound−lower bound).

**Table 2:** Relationships between baseline hippocampal–thalamic FA and symptoms at 12 months in UHR individuals only

	*Block I (baseline)*				*Block II (medication, age)*				*Block III (Thal*–*HPC FA)*			
	R^*2*^	*df*	*F*	P	*Δ*R^*2*^	*df*	*F*	P	*Δ*R^*2*^	*df*	*F*	P
									Right hemisphere FA			
Positive	0.454	1,24	21.87	0.001***	0.003	2,22	0.06	NS	0.076	1,21	3.58	0.036*
*β*	0.691***				NS,NS				0.295*			
									Left hemisphere FA			
Positive	0.455	1,24	21.87	0.001***	0.003	2,22	0.06	NS	0.069	1,21	3.21	0.044*
*β*	0.695***				NS,NS				0.284*			
									Right hemisphere FA			
Negative	0.288	1,24	11.09	0.003**	0.145	2,22	2.97	0.072^#^	0.011	1,21	0.438	NS
*β*	0.464*				0.417*, NS				NS			
									Left hemisphere FA			
Negative	0.288	1,24	11.09	0.003**	0.145	2,22	2.97	0.072^#^	0.006	1,21	0.226	NS
*β*	0.457*				0.412*,NS				NS			

Abbreviations: FA, fractional anisotropy; NS, not significant; Thal–HPC, thalamic–hippocampal; UHR, ultra-high risk.

Hierarchical regression was used and variables entered at each block are indicated. ^#^ *P*<0.1, *P<0.05, **P<0.01, ***P<0.001, one-tailed.
